# Feasibility Study: Improving Floor Cleanliness by Using a Robot Scraper in Group-Housed Pregnant Sows and Their Reactions on the New Device

**DOI:** 10.3390/ani9040185

**Published:** 2019-04-22

**Authors:** Peter Ebertz, Manuel Stephan Krommweh, Wolfgang Büscher

**Affiliations:** Institute of Agricultural Engineering, University of Bonn, 53115 Bonn, Germany; krommweh@uni-bonn.de (M.S.K.); buescher@uni-bonn.de (W.B.)

**Keywords:** animal welfare, livestock, mobile barn cleaner, modified cleaning robot, polluted surface areas of barns, hygiene of slatted floors, acceptance of moving objects in gestating sows, scoring of barn floors

## Abstract

**Simple Summary:**

A clean slatted floor in the animal housing is important for both hygienic and environmental reasons. In dairy farming, robot scrapers are used to support the floor cleaning process in the housing. As a result, the housing cleanliness is significantly improved. A robot scraper is equipped with various sensors and its own drive. A computer controls it. Taking into account barriers on the route (e.g., animals, walls, etc.), it moves at low speed over the housing floor and cleans it with or without the use of water. In this feasibility study, it has now been investigated whether such a robot scraper can also be used in pig farming. The faeces of pigs are drier than cattle excrement and thus more difficult to clean. Nevertheless, the robot scraper showed good cleaning results in this study. The sows accepted the presence of the robot scraper and were not disturbed by it. In summary, it can be stated that the cleanliness of the slatted floor in pig housings can also be increased by using a robot scraper. Since pigs are curious animals and investigate the robot scraper with their snouts, the technical configuration of the standard robot scraper should be slightly modified.

**Abstract:**

Successful pig farming needs the best conditions of cleanliness in the housings. The present study examined for the first time whether a robot scraper usually applied in dairy farming is usable in sow housings for cleaning the slatted floors and improving hygiene and thus animal welfare. For evaluating the suitability of the robot scraper with regard to the cleaning performance (polluted surface area and occluded slots), the whole housing area was divided into score-squares, which were individually scored at defined intervals. Selected excrement quantities removed by the robot were weighed. In order to assess the animals’ interactions with the robot scraper, their behaviour towards the device was observed. Although the faeces of pigs had a firmer consistency than bovine excrement, excrement quantities of up to 1.4 kg m^−2^ were almost completely removed. Even 6 h after the cleaning its effect was still visible. Dry-cleaning led faster to nonslip surfaces for the sows than wet-cleaning. Within half an hour of observation, up to 8.2 of 120 sows were occupied with the robot scraper, but without harming it. The use of robot scrapers in pig housings is recommended, although slight technical modifications should be made to the robot scraper.

## 1. Introduction

Modern livestock systems for pig husbandry have been used in the European Union for group housing of sows since at least 1st January 2013 [1]. A survey among German sow keepers confirms a trend towards larger animal groups. Forty-four percent of the sow keepers work with 16–40 sows per group and 22% work with groups of more than 40 sows in the waiting house. The most widely used housing system in this survey were large dynamic groups with electronic feeding stations [2]. Not less than 2.25 m² per sow may be offered at 6–39 sows per group and at least 2.05 m² per sow at a group size of more than 40 sows according to German legislation [1]. Especially in larger groups with more than 25 animals or more than 50 animals with electronic sorting or feeding stations, the animals’ functional areas such as ranges for activity, resting, feeding and excreting faeces are more clearly structured than in smaller groups [3]. This advantage in terms of living out the species-specific behaviour at the same time causes the pollution of faeces areas in housings, where the excrements are not deposited sufficiently through the slats of slatted floors [4]. According to German legislation, approximately 60% (depending on group size) of the floor in sows must be designed as lying area. Here, the slot rate of slatted floor must not fall below 15% [1]. As a rule, this is ensured with a slatted floor with maximum 18 mm slot width. The remaining approximately 40% of the floor area may also have higher slot rates, e.g., for the faeces area. In addition to poor drainage of excrements in faeces areas, further improvements in animal welfare will lead to feeding diets that are richer in crude fibre to saturate the pregnant sows. However this will inevitably lead to a different composition of the sow excrement [5,6,7] or slurry [8,9]. If the excrement is not removed mechanically or by hand, there is an increased risk of claw infections and ammonia emissions [10]. Wet conditions promote the degradation and the gas formation process [11]. Janssen and Krause assume that only 9% of the total odour emissions are caused directly by the animals, while 91% originate from polluted surfaces, especially from the barn floor [12].

In addition, polluted floors have an adverse effect on the sows’ claw health [13]. The accumulation of excrement on the concrete slatted floor increases the risk of slipping and claw infections. The excrement in the waiting house should be removed up to two times per day to effect rapid drying [14] and to ensure adequate surefootedness [15,16]. Australian studies showed that dry lying and activity areas are also desirable from the viewpoint of animal health and welfare. In the experiment the sows preferred significantly the dry housing areas. When the feed was supplied only in the wet region the sows went back immediately after the feed intake into the dry area [17].

The described problems of poor hygiene conditions [18,19], claw disorders [20] and an increased risk of ammonia emissions [21] in connection with polluted floors are already known from dairy farming. Because of the insufficient self-cleaning effect of slatted floors especially in the faeces areas, and to reduce the gas formation process it is necessary to remove the manure at regular intervals [22,23]. To handle this problem, automatic scrapers have been developed that clean the walking alleys at determined frequencies to improve their cleanliness [24]. Scrapers can significantly reduce the amount of manure on the barn floors [25]. In practical dairy cattle farming, these robot scrapers are already used in many housings. However, there is only few scientific literature on this topic, even in the field of dairy farming [24,26,27,28,29,30,31,32,33]. Ruud and Froknestad conducted a survey of dairy farmers (n = 52) who use robot scrapers in their housings. These were from the manufacturers either Lely or DeLaval, and almost all used the robot scrapers exclusively on slatted floors. Regardless of the brand, the farmers were consistently convinced of the cleaning performance, durability for cleaning and the time they had to spend for service. The only disturbances were when the robot “got stuck”. Here the farmers had to intervene from time to time so that the cleaning could continue. Altogether the equipment is expensive for the farmers, but they see advantages in improving the floor cleanliness and animal (claw) health [31].

Another study was investigated whether the use of a robot scraper could reduce emissions of climate-relevant gases as well as ammonia [32]. Significant reductions were achieved for methane (CH_4_), carbon dioxide (CO_2_) and nitrous oxide (N_2_O). For ammonia (NH_3_), no statistically verifiable reductions could be observed. The authors justify this, among other things, by the fact that mainly the solid fractions removed, while the liquid fraction is distributed even further in the housing. Furthermore, according to the authors, the surfaces do not have enough time to dry off. As a result, the liquid phase is distributed more widely and exposed to the air for prolonged periods [32].

Kai et al. also confirmed the statement that ammonia emissions in dairy farming cannot be reduced by automatic manure scraping. The automatic scraping frequency in dairy farms is between 6 and 12 times a day with both stationary scrapers and robot scrapers. No matter how often the scraper was used per day, the ammonia emissions could not be reduced significantly. Nevertheless, this of course reduced the amount of excrements on top of slatted floors and the walking alleys were cleaner than without or only cleaning once a day [33].

The use of robot scrapers in pig farming has not been described before. There is no scientific literature on this subject. For this reason, the obvious consideration was to transfer the solution for polluted floors from dairy to pig farming. The objective of the study was to investigate for the first time if a robot scraper from dairy farming can also be used in sow keeping, with the target of cleaning performance and acceptance by the animals in order to improve the hygiene conditions and thus the welfare of animals.

## 2. Materials and Methods

### 2.1. Animals and Housing

**Figure 1:**
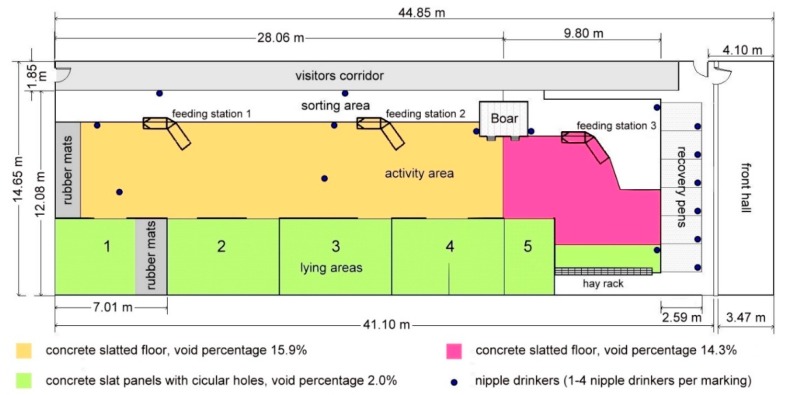
Situation during the experiment in the waiting house at the research centre ‘Haus Düsse’.

The study was carried out with a group of 120 pregnant sows at the ‘Centre of Research, Education and Training in Agriculture—Haus Düsse’ of the Agricultural Chamber of North Rhine-Westphalia in Bad Sassendorf in Germany. The whole sow herd included 220 sows during the experimental period, of which ~190 were the ‘Topigs’ breed and 30 were of Danish origin. After weaning of the piglets, the sows remained in the breeding centre for eight days. Three to four days after successful insemination, the sows were integrated into the large dynamic group in the waiting house, with three electronic feeding stations for 120 sows. The new feeding period started in the evening at 19:00 h. In the housing there was a larger space per animal (2.6 m² or 2.9 m²) than is currently required by law (2.05 m² [1]). The area of the waiting house was divided into 5 lying areas and a large activity area (Figure 1). Concrete slat panels with circular holes with a void percentage of 2.0% were installed in the lying areas. The circular holes in the panels had a diameter of 20 mm. The animals kept the lying areas very clean, which is why only a little urine had to drain through this minor slatted floor. Approximately three quarters of the activity area were equipped with a concrete slatted floor with 20 mm rectangular openings (void percentage 15.9%) for sows. Additionally, in the right part of the waiting house the last quarter of the activity area was equipped with a concrete slatted floor with 17 mm rectangular openings (void percentage 14.3%). Directly below and in front of the hay rack there was a row of concrete slat panels with circular holes like in the lying areas. The exact positions of the three different slatted floor types are shown in Figure 1. The areas marked with the three colours and the area of the rubber slat mats were completely at the disposal of the animals in the first part of the experiment. In the second part, lying area 3 was closed so that the available space per sow could be reduced, see also Chapter 2.3. This arrangement of the slatted floors was an advantage for the upcoming test, as three different slatted floor types could be tested simultaneously in combination.

The ventilation of the housing works with dripping ceilings for the incoming air and at the exhaust side with underfloor extraction. At high airflow volumes in the summer, exhaust air was additionally sucked through the upper floor. In addition to the electronic feeding stations for concentrated feed (diet for pregnant sows with 12.54 MJ ME and 13.38% crude protein), the sows could take hay ad libitum, available from the feeding rack (Figure 1, bottom right). Because of this, the excrement is very rich in fibre in contrast to pure feeding with concentrated feed.

### 2.2. Pretests and Adaptation of Animals

During pretests (not described in detail), data on sow activity was collected to define the best moving times for the robot scraper. There was no direct observation, as the animals could have felt influenced by humans. Therefore the observation was via video technology with three cameras (Sec-Cam 31 from König Electronic GmbH; Reichelsheim, Germany and monitoring software package ABUS VMS basic from August Bremicker Söhne KG; Wetter, Germany). Because the feeding started in the evening, the lowest sow activity was observed throughout the day between 8:00 and 19:00 h. 

**Figure 2:**
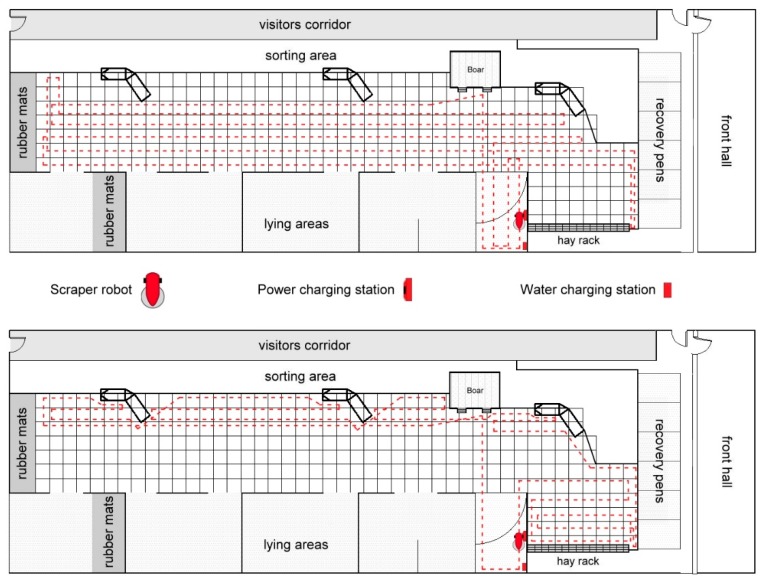
Score-squares for evaluation of cleaning performance and routes of the robot scraper determined in the activity area. Route 1 (above) and route 2 (below) were executed one immediately after the other. The icons of the scraper robot and charging stations originate from Lely Holding S.à.r.l.

Subsequently, the data of the “general contamination of the housing without robot scraper” were collected on three randomly selected days before start of the testing phase with the device. The same method with the score-squares as shown in Figure 2 and described in Chapter 2.3.1 was used for this purpose. In addition, the locations of the faeces areas were identified in order to plan the routes (Figure 2) for the autonomous robot scraper Lely ‘Discovery 90 SW’ (described in detail in Chapter 2.3).

After the first tests, modifications to the standard robot scraper were necessary. The robot scraper could not navigate satisfactorily in the housing area. To solve this problem, a second ultrasonic sensor was installed. Both ultrasonic sensors were screwed on a 750-mm-high antenna. After this modification, the robot scraper could detect the walls for orientation and not the lying animals. Further improvements to the navigation were made with a recalibration and the transferring of the power and water charging stations.

Based on these results, the routes in the housing and the cleaning frequencies of the robot scraper were established for the main experiment (Figure 2). A distribution of routes had to take place because a certain number of commands could not be executed in the programming software of the robot scraper. 

In order to ensure that conditions were as is usual in practice, the housing was deliberately not thoroughly cleaned with a high-pressure cleaner before the robot scraper was used.

After this first testing period the animals had to get familiar with the robot scraper. First, the robot scraper was placed in the sow group three times a day (30 min each) for three days. Then the sows could familiarise with the driving robot scraper for two weeks. The operating times of the robot scraper were the same as in the following main experiment.

### 2.3. Main Experiment

For the main trials a battery-driven robot scraper named ‘Discovery 90 SW’, manufactured by Lely Holding S.à.r.l (Maassluis, the Netherlands), with a water spray feature, was available. It was initially a standard robot scraper also used in dairy farming. When the robot scraper moves forward, a rubber lip under the front ring pushes the excrements down through the slats. In addition, the water spray feature allows the moistening of encrusted excrements, which can then be removed during the next cleaning run. The two spraying nozzles together need 1 l min^−1^ of water, so the 30-litre tank is enough for a cleaning period of half an hour [34]. In this time the scraper robot cleans an area of approximately 210 m². The manual operation works via a wired remote control to determine the driving routes and the moving times of the robot scraper. The robot scraper navigates the housing with two electric wheel motors, a gyroscope and in its original condition with an ultrasonic sensor on the lower left side. The cleaning speed can range between 9 and 18 m min^−1^ [35]. In the trial, a slow speed was chosen in order to protect the animals. On long straight directions, the robot scraper’s speed was 11 m min^−1^, but the turning times reduced this to an average cleaning speed of 9 m min^−1^. The robot scraper weighs 340 kg; length = 1.365 m, width = 0.88 m and height = 0.60 m [35]. Compared to the competitors, this robot scraper is somewhat smaller and seemed ideal for use in a pig housing, which is usually smaller than large dairy cattle housings.

There was a daily cleaning iteration at 8:30 h and at 15:00 h in two periods of 20 days overall between March and April and May and June 2014. In the first ten test days, the robot scraper cleaned in the morning without the water spray feature and in the afternoon with the water spray feature. The available space per animal was 2.9 m^2^. In the following ten test days, the trial was reversed with water spraying in the morning and dry-cleaning in the afternoon. With the reduction of one part of the pen (lying area 3 was closed, cf. Figure 1), each sow had 2.6 m^2^ available in order to get closer to the conditions that are customary in practice. Each time, the robot scraper cleaned only the activity area, as the lying areas were usually free of excrement and could serve the sows as a retreat from the robot scraper.

#### 2.3.1. Validation of the Polluted Surface Area and Occluded Slots as well as the Determination of Pollution Intensity

To determine the initial pollution, the cleaning efficiency and the recontamination after the cleaning, the whole activity area (210 m^2^) was divided into score-squares. Each score-square was 0.8 m × 0.8 m and was recognisable by the installed slatted floor elements: One slatted floor element was 0.4 m x 1.6 m; two elements next to each other together form two score-squares with the above dimensions. The 312 score-squares of activity area are also shown in Figure 2. The cleaning runs started every day at 8:30 and 15:00 h. The squares were rated immediately before the cleaning runs (8:30 and 15:00 h), immediately after (9:00 and 15:30 h) and at intervals of two (11:00 and 17:30 h), four (13:00 and 19:30 h) and six hours (15:00 and 21:30 h) after cleaning the surface area. The rating was carried out by the same person over the entire trial period and 180 score plans from different periods were created (2x10 days x 9 plans per day). The pollution was differentiated into the pollution of the surface area (1 = green = dry/clean; 2 = yellow = wet; 3 = red = polluted; 4 = black = wet and polluted) and the percentage of occluded slots in the slatted floor (a = white = 0–25% blocked; b = light grey = 26–50% blocked; c = dark grey = 51–75% blocked; d = black = 76–100% blocked). For the entire scoring the average pollution or the average proportion of occluded slots of one score-square was always considered and noted.

Before and after cleaning, the excrement weights on individual slats panels were weighed two times daily in main experiment to find out what percentage of the excrement could be removed by the robot scraper. For this purpose, two adjacent, uniformly soiled slat panels were selected in the entire activity area before cleaning, and the manually scraped excrements of one of the two slat panels were weighted in plastic bags on scale. The scale used had a graduation of 0.02 kg and the lowest displayable weight above zero was also 0.02 kg. A distinction was made between the concrete perforated panels with circular holes, the slat panels with 17 mm rectangular openings and the slats panels with 20 mm rectangular openings, as the excrement quantities obviously differed depending on slatted floor type. All three types of slatted floors could be found in the activity area (cf. Figure 1). After cleaning, the remaining excrements of the adjacent, previously left polluted slat panel was weighed to measure the ‘cleaning effect’.

#### 2.3.2. Determination of Contacts between the Animals and the Robot Scraper

In order to record the reactions of the animals to the robot scraper, three simple behavioural tests with the ‘novel object’ robot scraper were performed.

The first test was the ‘moving novel object test’. The number of sows occupying themselves with the moving (30 min per cleaning run) robot scraper was counted to determine if the animals might damage the robot scraper while cleaning. A contact was counted as a contact with the robot scraper when the snout of a sow touched part of the robot scraper in a targeted manner; additionally, the number of sows that have been touched or almost touched by the moving robot scraper and that changed their places immediately before or after this contact was observed.

The second test was the ‘standing novel object test’. The standing robot scraper was examined to construct the worst case, i.e., to find out what happens later if the device potentially gets stuck when the farmer is not in the housing. A defect was simulated to find out whether the robot scraper could be damaged or rendered unusable by the animals’ play instinct within half an hour, until the farmer is present. For that two times at all days of the main experiment, every 30 min immediately after the cleaning runs in the morning (9:00 h) and afternoon (15:00 h) the robot scraper was positioned in the middle of the animal group in the activity area (n = 2x20). A contact was counted as a contact with the robot scraper when the snout of a sow touched a part of the robot scraper in a targeted manner.

A third survey looked at the attraction of different individual protruding components on the standing robot scraper. The animal was considered to be engaged if it specifically moved towards the specific component or head and palpated the component with its snout or bit into it. A contact was counted as a contact with one component until the corresponding sow was occupied with another component or completely turned away from the robot scraper. 

All these observations were monitored live via cameras (same technology as described in Chapter 2.2) from the front hall of the housing during the 30-min cleaning run and the 30-min standing time to enable the animals to show their natural behaviour and prevent them being influenced by any human being present. Data was collected from Monday to Thursday. The animals were familiar with the robot scraper because of the 2-week adaption period before the trial. New sows (groups of 8–10 animals every week) were introduced every Friday morning in the dynamic group. That was the reason why there was no collecting of data. Next collecting starts on Monday morning, so that these small groups of new sows had minimum of 3 days for adaption to the robot scraper.

### 2.4. Statistical Analysis

The measured data were statistically analysed with IBM SPSS Statistics 24. The descriptive statistics involved the sample size, mean value, standard deviation, minimum and maximum. For an improved and clear presentation, charts or tables were created for all parts of the study. Given the particularly small sample sizes, the tests were conducted on a normal distribution using the Kolmogorov–Smirnov test at relevant points. When there was a normal distribution, a *t*-test was chosen to determine significant differences. Without normal distribution, the Kruskal–Wallis test was used. In which case which test was used is noted in the results. All tests used were based on a level of significance of 0.05. 

## 3. Results

### 3.1. General Contamination of Slatted Floor in The Experimental Housing without Cleaning

**Figure 3:**
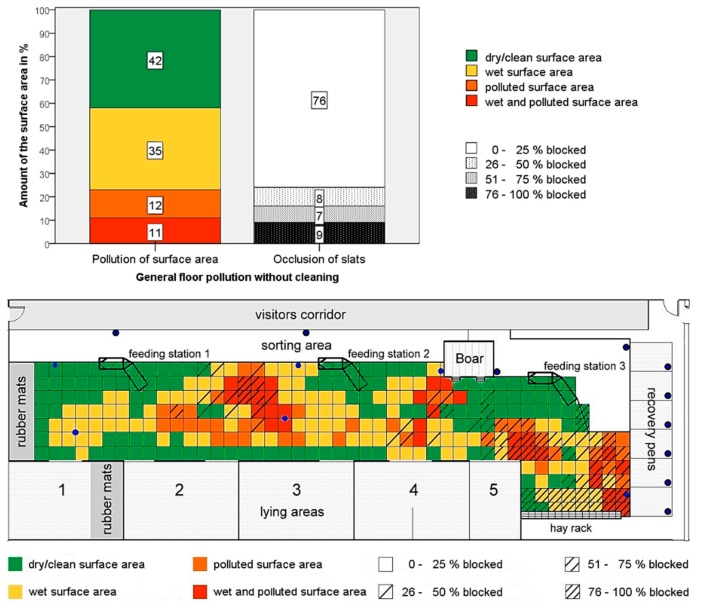
‘Representative floor pollution’; Above: Average pollution without cleaning over three randomly selected days before the start of the experiment. Below: Housing plan with distribution of floor pollution from 24.02.2014.

Before the robot scraper was first used in the barn, the floor area was scored at three randomly selected days before the start of the first tests. Compared to the other two days, the data of the day 24.02.2014 can be regarded as “representative floor pollution”. Therefore, these data were illustrated in the housing plan of Figure 3. Above this, the mean values of the three days recorded are shown.

The superficial pollution is particularly evident in the area of the nipple drinkers and in the slatted floor types with smaller void percentage. The area at the boar pen (nipple drinker), the corner area in front of the lying area 5 (lower slot width, 17 mm) and the area in front of the hay rack (lower void percentage) are also polluted. Slightly less contaminated (‘polluted’) are the edge areas of the heavier pollution and the area towards the left feeding station. Slight pollution (‘wet’) can be seen in the transition areas and at the other nipple drinkers. The edge areas are predominantly clean (‘dry/clean’) without drinking places.

The amount of occluded slots is similar (Figure 3). Especially in the slatted floors with a lower void percentage in the area of the hay rack, the slats are more closed than in the slatted floors with 20-mm rectangular openings. With the exception of the hay rack area, the slats installed at the edges are largely free. The slatted floors around the nipple drinkers are also free. An exception is the floor around the nipple drinker in front of the hay rack. 

Forty-two percent of the activity area is kept completely clean by the animals. Thirty-five percent are only slightly polluted. Just under a quarter of the entire activity area can be described as very polluted, with 12% being polluted and 11% both wet and polluted. The total polluted area of 23% corresponds to just less than 83 m² of the housing floor available for the animals. With 120 animals, this results in a polluted housing area of 0.7 m² sow^−1^. Figure 3 also shows the amount of occluded slots. Approximately three quarters of all slats were ‘free of excrement’.

### 3.2. Results of the Cleaning Performance

**Figure 4:**
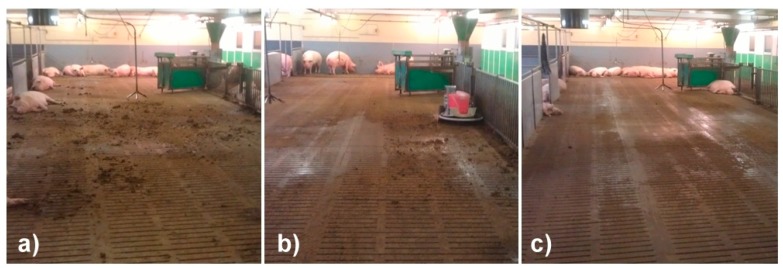
Condition of floor area before (**a**), during (**b**) and after cleaning (**c**) in experimental housing.

During the two weeks of adaptation, the animals accepted the robot scraper very well. The excrement was removed within the 30-min cleaning runs (Figure 4). The robot scraper was able to drive under the free-hanging nipple drinkers without any problems because they were hanging exactly above the height of the robot (0.8 m). 

**Figure 5:**
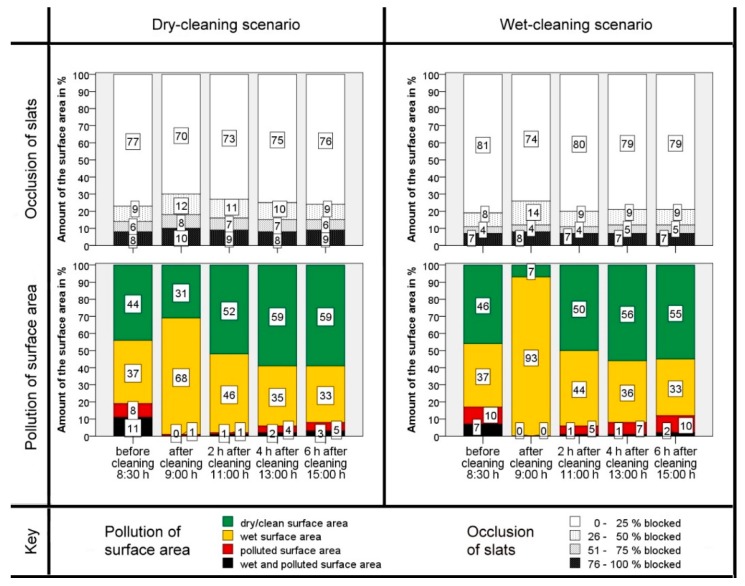
Results of the cleaning performance of the robot scraper before cleaning and at different intervals after cleaning for the cleaning runs in the morning (Start: 8:30 h): Proportion of occluded slots (above) and degree of polluted surface area (below) for both cleaning scenarios, dry-cleaning (left) and cleaning with the use of water (right). The numbers in the bars indicate the respective percentage of the total floor area of the experimental compartment.

**Figure 6:**
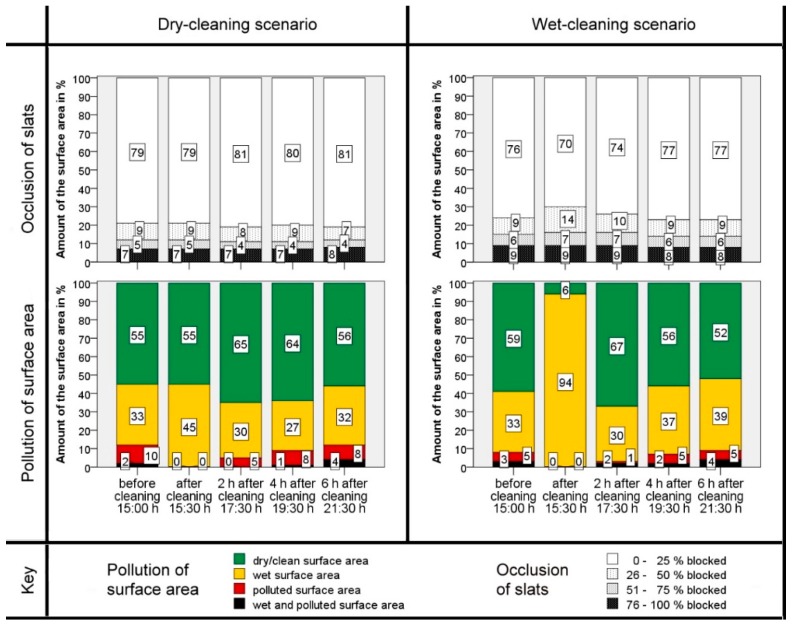
Results of the cleaning performance of the robot scraper before cleaning and at different intervals after cleaning for the cleaning runs in the afternoon (Start: 15:00 h): Proportion of occluded slots (above) and degree of polluted surface area (below) for both cleaning scenarios, dry-cleaning (left) and cleaning with the use of water (right). The numbers in the bars indicate the respective percentage of the total floor area of the experimental compartment.

The cleaning results and the recontamination over the time after the cleaning runs are shown in Figure 5 and Figure 6. These show the score for the morning and afternoon cleaning runs, differentiated by wet- or dry-cleaning scenarios.

In all scenarios the contamination was significantly (*t*-test, *p* < 0.05) reduced by the cleaning. The amounts of the ‘polluted’ and ‘wet and polluted’ areas decreased to a minimum after cleaning. The amount of wet slatted floor increased after cleaning with water (93%, Figure 5) more than in the dry-cleaning (68%, Figure 5) scenario. This difference is significant (*t*-test, *p* < 0.05). The same scope can be observed also in Figure 6. In comparing both dry-cleaning scenarios, the amount of the ‘wet’ surface area increased more strongly during the cleaning runs in the morning. The difference is significant as well (*t*-test, *p* < 0.05). The initial pollution is higher in the morning than in the afternoon (*t*‑test, *p* < 0.05).

Two hours after cleaning, the ratio of the ‘wet’ areas decreased. At the same time, the proportion of the ‘dry’ areas and also the ‘polluted’ and ‘wet and polluted’ surface areas increased again. This development continued constantly until six hours after cleaning. The higher initial pollution in the morning (8:30 h) before cleaning ensured that, even six hours after cleaning (15:00 h), the degree of initial pollution had not yet been reached again. The two t-tests carried out for this purpose confirm the significant difference (*p* < 0.05). At the cleaning in the afternoon the initial pollution was lower (15:00 h), and six hours after cleaning (21:30 h), the final pollution was comparable with the initial pollution in the afternoon as the difference is not significant (*t*-test, *p* > 0.05).

In addition to the polluted surface area, the percentage of occluded slots in the slatted floor was observed. The main part of the slats (more than 70%) was always clean and free of excrement. In the study the amount of totally blocked or smeared slots increased right after the cleaning runs. This difference is significant between the dry- and wet-cleaning scenarios in the afternoon (*t*-test, *p* < 0.05), but this trend could just not be statistically verified with the data of the morning cleaning run (*t*-test, *p* > 0.05) although the data look similar. Two and four hours after cleaning the proportion of blocked slots reverted continually to the initial level and barely changed afterwards. 

The robot scraper cleaned in the main experiment an area of 210 m² within half an hour and had a total water consumption of 30 l, if it cleaned with water. Per cleaning run and square metre, this results in a water consumption of 0.13 l m^−2^. Per sow, this would be 0.25 l d^−1^. In our case, there is a total of approximately 11 m³ y^−1^ or 92 l sow^−1^ y^−1^ more water or slurry when there is one cleaning run ‘with water’ per day. In order to quantify the cleaning performance of the robot scraper, the excrement masses of the three slatted floor types were randomly examined before and after the cleaning runs. In all cases the relative zero point of 0.02 kg was reached after the cleaning (Table 1). The scales showed a weight of 0.02 kg both for an empty bag and for the bags including the amount of excrements after the cleaning runs. The quantities of the remaining excrements residues could therefore not be determined exactly due to their low mass, but were always close to zero. Therefore, it can be seen that the robot scraper removed almost all the excrements. In addition to the cleaning performance, Table 1 shows that, on average, all slat floors showed more excrement before cleaning in the morning than in the afternoon. They differ significantly (*p* < 0.05, Kruskal–Wallis test). The effect of the morning cleaning is, therefore, still visible six hours later. Equally significant with *p* < 0.05 (Kruskal–Wallis test) is the difference between the excrement amounts before cleaning depending on slatted floor type. Regardless of the time of cleaning (morning/afternoon), the highest average amounts of excrements were found on the slat panels with circular holes, the second highest average amount on the slat panels with 20-mm rectangular openings and the lowest average amount on the 17-mm slat panels with rectangular openings.

### 3.3. Sows’ Interactions with the Robot Scraper

**Figure 7:**
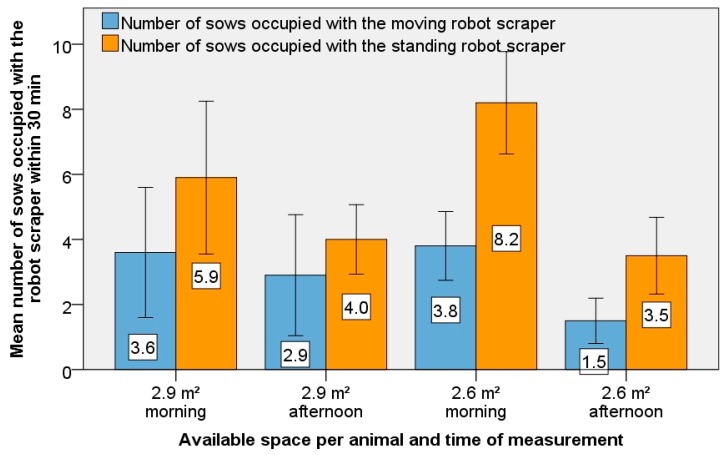
Representation of the average number of sows occupied with the moving or standing robot scraper in the morning and afternoon, with different space of 2.9 square metres per sow (left) or 2.6 square metres per sow (right).

Figure 7 compares the number of sows who occupied themselves with the robot scraper when either moving or standing. During the moving or standing time of 30 min a mean number of between 1.5 and 8.2 sows showed interest in the robot scraper. That was a maximum of 7% of the sows in the entire experimental housing. In the morning, there was a significantly higher interest in the robot scraper than in the afternoon (*t*-test, *p* < 0.05). The available space per animal had no significant effect on their interest in the robot scraper (*t*-test, *p* > 0.05). All trials showed that the standing robot scraper was much more attractive for the animals than the driving robot scraper. At the higher available space per animal (2.9 m^2^ per sow) the sows were 1.5 times more interested in the standing than in the moving robot scraper (Figure 7; *t*-test, *p* < 0.05). At 2.6 m² per sow, the number was twice as high. The difference compared to the moving robot scraper is significant (*p* < 0.05). 

**Figure 8:**
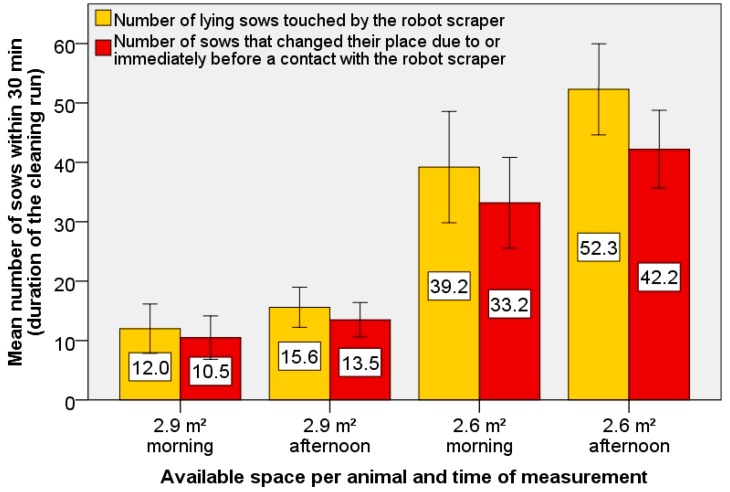
Representation of the average number of sows affected by the robot scraper during the cleaning process (yellow) and the average number of sows who changed their places as a direct result of the contact or immediately before the contact (red), separately shown for the observations in the morning (8:30–9:00 h) and afternoon (15:00–15:30 h) with different space of 2.9 square metres per sow (left) or 2.6 square metres per sow (right).

Figure 8 shows the mean number of sows that the robot scraper touched while cleaning and of those who changed their lying places immediately before or after this contact. The difference in bar scales gives the number of sows that were touched by the robot scraper but did not move from their lying place. In these cases, the robot scraper moved around them to continue the cleaning route. 

The variants show significant differences. At the greater space of 2.9 m² per sow, the animals were less disturbed by the robot scraper. This general distinction between sows touched in more and less space per animal is significant (*p* < 0.05). At less space, the difference between the absolute number of sows in the morning and afternoon cleaning-run is higher.

This difference between the number of sows touched in the morning and afternoon at 2.6 m² per sow is significant (*p* < 0.05). With the greater space of 2.9 m² this tendency can be seen from the mean values as well, but there is no significant difference (*p* > 0.05). The difference between the sows affected and the sows that then changed their place is higher at 2.6 m² per sow, so more animals stayed in the same places after being touched by the robot scraper. It can be stated that no animal was disturbed or violated by contact with the robot scraper. If this had been the case, the trial would have been stopped immediately.

**Figure 9:**
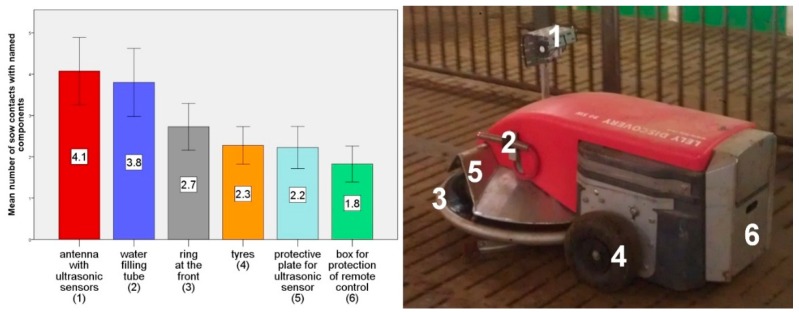
**Left:** Average number of sow contacts with individual components of the robot scraper in 30-min observation period. **Right:** Components on the robot scraper.

The numbers of animal contacts with particular components of the robot scraper were also observed. An average of from 1.8 to 4.1 sow contacts was counted in half an hour (Figure 9). More protruding components like the water filling tube were of significantly greater interest for the animals than the less protruding parts like the tyres (*p* < 0.05). In all the trials attention was paid to whether an intervention was needed to protect the robot scraper from being damaged by the sows, but it can be stated that this was not necessary. During the experimental periods, the robot scraper was not attacked by the animals.

## 4. Discussion

### 4.1. Floor Condition without Cleaning

Polluted slats are found especially around nipple drinkers. Meyer and Jahn confirm that overflowing drinking water increases the probability that these zones will be used as excrement areas [4]. This statement also explains why no pollution was found around the nipple drinker to the left of the third feeding station (cf. Figure 3). Here, there is a drinking trough with a large bowl in which overflowing water is collected and thus cannot reach the floor. In addition to the drinking zones, polluted and occluded slats were also found in the experiment in areas with a lower void percentage. According to Weber and Meyer [36], closed areas in the activity area are always a problem, as they are often used by the animals, especially at higher temperatures, as a surface for fecalising and urinating, in order to create ‘wallows’. Pen design and ventilation also influence the arrangement of the functional areas of pigs.

An excrement area of 0.7 m² per animal (‘polluted’ plus ‘wet and polluted’ in 120 sows) could be calculated in this experiment. The situation is aggravated by the fact that the experimental housing is understaffed and thus an even larger area per animal is available (2.9 or 2.6 m² sow^−1^). As a result, the excrement areas become larger, as the excrement is not sufficiently drained through the slats.

### 4.2. Valuation of the Cleaning Performance

After cleaning with water, the amount of ‘wet’ surface area increased significantly more than after the dry-cleaning scenario. The water spray feature can help together with a high scraping frequency to avoid incrustations of excrement on the floor. Nevertheless, pure dry-cleaning is more advantageous in sow-keeping as it enables a faster, dry clean and, additionally, a nonslippery floor surface, as required by Pluym et al. [37]. There is also no additional water consumption. Per cleaning run per day, 11 m³ more slurry is produced at the end of the year. If there are several cleaning runs “with water” per day, correspondingly more slurry storage volume has to be provided which in turn is associated with follow-up costs.

The automated cleaning leads easy to improved cleanliness, less slipping and a lower risk of infections for the animals [10,13,14]. Wiedmann et al. recommend cleaning several times a day in the waiting house [14]. An autonomous robot scraper can achieve a further improvement of hygiene without needing additional working time for the actual cleaning in the future. On the other hand, times for maintenance and care of the robot scraper as well as expenses for possible malfunctions must be taken into account. According to Ruud and Froknestad [31], who conducted a survey at dairy farmers with robot scrapers, the robots suffered an involuntary disorder requiring intervention approximately every 10 days (at higher cleaning frequencies than tested in this study). Normally the farmers there just had to restart the robot scrapers. Nevertheless, by automating this work it is possible to save valuable working time in larger sow herds. The improvement of environmental hygiene is in fact becoming increasingly important [24] not only in dairy farming, but also in sow management. The farmers described in Ruud and Froknestad were very satisfied with the job by the robot scrapers also with regard to claw health [31].

In all cases, the excrement was removed to a minimum regardless of the slatted floor type (cf. Table 1). The results also show that in spite of the firmer faeces and the narrower slots (max. 18 mm for fattening pigs or in the lying area for sows or 20 mm for gilts and sows [1]) in the slatted floor in sow-housing than the slatted floors in dairy farming (35 mm [38]), a very good cleaning performance could be obtained. The faeces were removed almost completely at all places where the robot scraper passed over. Table 1 shows exemplary pollution of slat elements and does not allow any conclusion to be drawn about the total quantity of cleaned excrement in the entire housing. Nevertheless, it is easy to see how much excrement the device is capable of removing at the selected ’hot spots’.

Especially immediately after cleaning, a larger amount of smeared slots was expected. However, at least 70% of the slots remained permanently free. The previously feared permanent blocking of the slots caused by the manure scraping could not be observed during the investigation. The deposition of sow excrement, herein additionally rich in fibre due to the supplementary hay feeding, through the slats by the commercially available robots therefore also works satisfactorily as in dairy farming. 

No statements could be made in this study with regard to ammonia emissions. Chiumenti at al. [32] and Kai et al. [33] could not detect any significant reduction in ammonia emissions in dairy housings with automatic scraping several times (6–12 ^−1^) per day. The results in this study show that with scraping only twice a day, the floor area in sow housing dried quite quickly after scraping. Further studies would have to clarify whether this could have a positive effect on ammonia emissions.

### 4.3. Sows Interactions with the Robot Scraper

The sows’ interest in the robot scraper was greater in the morning than in the afternoon. This is related to the higher activity level of the sows in the morning, which was measured in pretests and is related to the feeding management and the refilling of the hay rack every morning [39,40]. Contrary to expectations, only a few sows interacted with the robot scraper. It was expected that the movement of the robot scraper would animate the sows to interact or play with it. The opposite happened, and the standing robot scraper was found to be of greater interest for the animals.

Similar to Sagkob et al. [29] in dairy farming, the sows in this study took very well to the robot scraper after a short familiarisation phase of two weeks and they usually avoided it during the cleaning runs in the main experiment. The number of sows touched by the robot scraper was higher in the afternoon. This can equally be explained by the activity level of the animals. Under the prevalent conditions in this housing, they were more active in the morning. They were therefore more able to avoid the robot scraper. In the afternoon, they were rather inert and had a distinct resting period. Similar to the studies on dairy cows by Stülpner et al. [30], the sows that were touched by the robot scraper seemed to look for a quieter retreat, in this case the lying areas outside the cleaning routes of the robot scraper. The animals adapted very well to the use and the operating times of the robot. In dairy cows, several authors were able to demonstrate that the stress level for the animals when using a robot scraper is very low and animal welfare is not impaired [26,27,28]. Nevertheless, Ruud and Froknestad noted that injuries can occur very sporadically [31]. Here the manufacturers should still work on the safety systems of the robot scrapers.

Since pigs are usually more active than cattle and show a stronger interest especially in new housing equipment or manipulable material [41], the attraction of different components of the robot scraper was measured. Especially the more protruding components aroused the interest of the animals. With further development of robot scrapers for pig housing, an encapsulated design should be considered to avoid damage by the animals, although in the trials no problems of this nature occurred.

## 5. Conclusions

The cleaning performance of the robot scraper can be described as very good despite the lower slot widths and the firmer excrement consistency in sow keeping. In any case, the floor area was significantly less polluted than without automatic cleaning. The use of a robot scraper could improve the cleanliness of pig housings. Wet-cleaning helps to remove or avoid incrustations, but pure dry-cleaning is preferable if possible, as no additional water is used and the floors dry off faster after cleaning. Further experiments would have to be carried out to find out whether, in contrast to dairy farming, ammonia emissions in pigs could perhaps be reduced if the floor areas dried more quickly. 

No animal was disturbed or violated by contact with the robot scraper. Contrary to what was assumed, the standing robot scraper was more interesting for the animals than the driving device. The concern that the robot scraper could be damaged by the animals’ play instinct was not confirmed in the trials. Further tests are necessary under which conditions the robot scraper can be used. Next, a robot scraper should be tested in a housing that has less space per sow than in this study or the legally required space per sow (2.05 m² depending on group size). The standard robot scrapers from dairy farming need to be modified to achieve better navigation in pig housings. In our opinion, at least a second ultrasonic sensor is indispensable to improve navigation. Further optimisations with regard to an easier navigation would be desirable. Furthermore, robot scraper for sow husbandry should have a more encapsulated design with fewer protruding components to reduce the attraction for the sows.

## Figures and Tables

**Table 1:** Quantitative cleaning performance: Removed excrement quantities per m² and type of slatted floor, differentiated according to the cleaning runs in the morning or in the afternoon.

Quantitative Cleaning Performance after the Cleaning Runs in the Morning					
Slats panels with circular holes (Diameter 20 mm)		Slat panels with 17-mm rectangular openings		Slat panels with 20-mm rectangular openings	
BC	AC	BC	AC	BC	AC
1.42 kg m^−2 a^	0.02 kg m^−2^	0.64 kg m^−2 a^	0.02 kg m^−2^	0.98 kg m^−2 a^	0.02 kg m^−2^
BC	AC	BC	AC	BC	AC
0.61 kg m^−2 b^	0.02 kg m^−2^	0.38 kg m^−2 b^	0.02 kg m^−2^	0.47 kg m^−2 b^	0.02 kg m^−2^

BC = Before cleaning; AC = After cleaning; ^a,b^ show significant differences of the amounts of excrements after the morning and afternoon cleaning runs.
